# Species diversity of fish at the Wuzhizhou Island, South China Sea, based on environmental DNA

**DOI:** 10.3897/BDJ.12.e127120

**Published:** 2024-06-14

**Authors:** Chongzhao Wang, Zhenhua Ma, Kun Cao, Xin Wang, Rui Xi, Ting Jiang, Rui Yang, Yingchun Xing

**Affiliations:** 1 Hainan Fisheries Innovation Research Institute, Chinese Academy of Fishery Sciences, Sanya 572024, China Hainan Fisheries Innovation Research Institute, Chinese Academy of Fishery Sciences Sanya 572024 China; 2 Resource and Environmental Research Center, Chinese Academy of Fishery Sciences, Beijing 100141, China Resource and Environmental Research Center, Chinese Academy of Fishery Sciences Beijing 100141 China; 3 National Demonstration Center for Experimental Fisheries Science Education, Shanghai Ocean University, Shanghai 201306, China National Demonstration Center for Experimental Fisheries Science Education, Shanghai Ocean University Shanghai 201306 China; 4 Tropical Aquaculture Research and Development Center, South China Sea Fisheries Research Institute, Chinese Academy of Fishery Sciences, Sanya 572018, China Tropical Aquaculture Research and Development Center, South China Sea Fisheries Research Institute, Chinese Academy of Fishery Sciences Sanya 572018 China

**Keywords:** marine ecosystem, occurrence of fish taxon, distribution, sequences of MOTUs, the South China Sea

## Abstract

**Background:**

Wuzhizhou Island (WZZ) is located in Haitang Bay in the northern region of Sanya, Hainan Island. The sea area surrounding WZZ represents a typical tropical marine ecosystem, characterised by diverse and complex habitats. Therefore, there is a rich variety of marine fish species at WZZ. The marine ecosystem of WZZ was seriously destroyed initially in the 1970s-1980s and recovered in the 1990s, then constructed as the first national tropical marine ranch demonstration area of China in 2019. As fish is an important high trophic vertebrate in the marine ecosystem, understanding the composition and distribution of fish species could help us to recognise the status of the ecosystem of WZZ and supply scientific data for construction of the national marine ranch demonstration area. This study used eDNA technology to investigate the composition of fish community surrounding WZZ and provided a scientific basis for realising and protecting the marine ecosystem of the South China Sea.

**New information:**

The WZZ is an offshore island in the South China Sea, harbouring abundant marine fish resources. Although previous research investigated fish species of WZZ, the data were, however, still incomplete due to limitation of sampling methods and survey seasons. In this study, we intended to take advantage of eDNA and supplement data of fish species at WZZ as much as possible. Based on eDNA, this study provided the data on 188 fish species (including nine undetermined species denoted by genus sp.) belonging to 17 orders, 63 families and 124 genera and they were the more comprehensive records of fish species surrounding WZZ. In addition, the information on Molecular Operational Taxonomic Units (MOTUs) for taxon identification was also provided, aiming to contribute to the establishment of a specific eDNA taxon database for fish of the South China Sea. This study included two datasets, which were occurrences of fish taxa at WZZ, as well as MOTUs sequences and geographical coordinate information of sampling sites. The “fish taxon occurrences” dataset presented records on taxonomic, distribution and habitat conditions of 188 fish species detected using eDNA, as well as the latitude and longitude information of the sampling sites, the "MOTUs information" dataset provided the MOTUs sequences, source of sequences, abundance of sequences for 188 fish species, also included the species matched in NCBI and the best NCBI BLAST sequence similarity.

## Introduction

The Hainan Island is located in the South China Sea, covers a land area of 35,400 km^2^, as well as having a vast sea area of nearly 2 million km^2^ and 1,618 km of coastline (Lin et al. 2022). The coastal area of the Hainan Island is rich in habitats such as rocky, coral reefs, gravel as well as sandy and muddy substrates (Liu 2013), which breed complex biological communities and high biodiversity. The marine fish, as an important part of the marine ecosystem, plays a key role in material cycling and energy flow, as well as maintaining the ecological functions (Ault et al. 2022, Ziegler et al. 2023). Therefore, the study of fish diversity in specific marine areas is essential for monitoring and evaluating the status of the ecosystem and it is able to offer valuable insights for marine ecological restoration and protection (Zou et al. 2020).

The Wuzhizhou Island (WZZ) of Sanya City is an outlying island of Hainan Island, with an area of 1.48 km^2^ and 5.7 km of coastline, charmingly resembling an irregular butterfly in shape (Luo et al. 2023). WZZ is located at Haitang Bay, affected by the tropical monsoon climate (Yin et al. 2023) and its northern coast is sandy and the southern coast is mainly rocky (Xu et al. 2020). Consequently, the water area at WZZ is distributed by abundant marine fish resources. With the exploitation of WZZ over the past decades, its ecosystem was significantly damaged by human activities, such as overfishing, land reclamation, coral mining, dredging, aquaculture expansion and pollution (Zhang et al. 2006). However, these negative effects began to be eliminated in 1997 when the Wuzhizhou Company initiated tourism development on the Island (Huang et al. 2020). In 2010, WZZ embarked on a marine ranch construction project, leading to the recognition of it as the first national-level marine ranch demonstration area in Hainan Province by 2019 (Li et al. 2015, Wang et al. 2021, Luo et al. 2023). At present, the studies on fish diversity of WZZ are to be conducted, aimed to understand the status of fish species diversity and evaluate the effectiveness of the sea ranch.

The environmental DNA (eDNA) has been known as a useful tool to detect aquatic and semi-aquatic species by extracting DNA from environmental samples such as water and sediment (Rees et al. 2014, Pedersen et al. 2015). Indeed, this technique has been widely used to monitor marine fish species, especially tropical marine biodiversity (Gelis et al. 2021, Mathon et al. 2022, Xi et al. 2022, Zhang et al. 2023). Compared to major traditional marine monitoring methods, such as fishing nets and diving, eDNA could provide more species information and less harm to the wildlife (Jerde et al. 2011, Sales et al. 2021). This study applied eDNA technology to provide valuable data that enrich the database of tropical marine fish, which would be a benefit for protecting the ecosystem of the South China Sea.

## Sampling methods

### Study extent

The sea area surrounding WZZ is located in Haitang Bay, coastal Sanya City, Hainan Province, China.

### Sampling description

**Figure 1.**
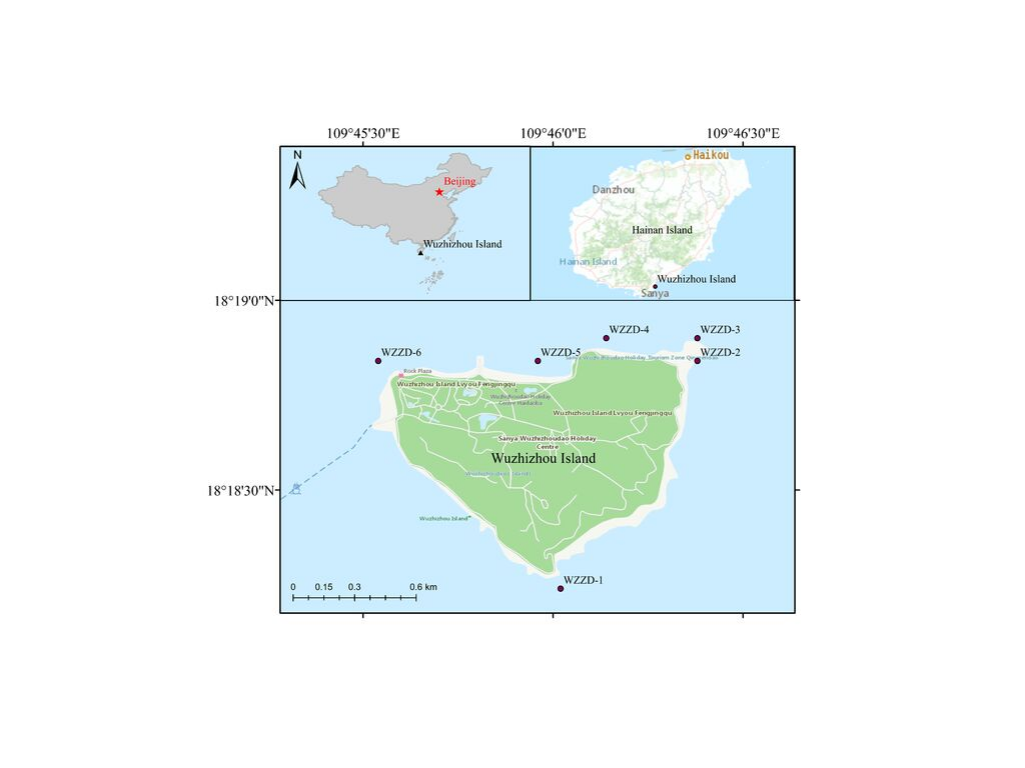
Location of eDNA sampling sites.

**Figure 2.**
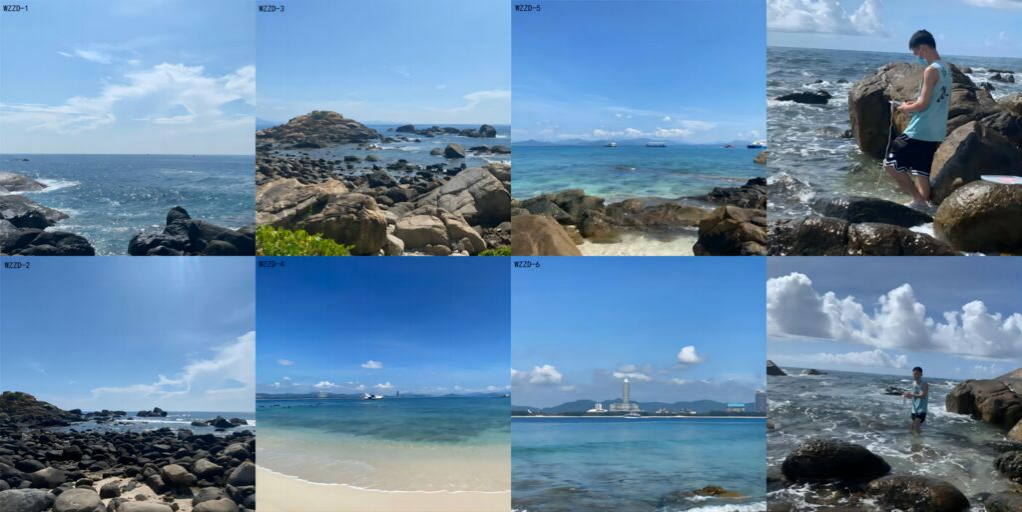
Photographs of marine habitats and field sampling around WZZ.

The sampling protocols referenced our previous research (Xing et al. 2022) and the main steps were as follows: the shallow seawater samples were collected using a 3 l hydrophore at six sampling sites in sea area surrounding WZZ in June 2022, as well as January and September 2023, the Hainan Province, China (Fig. 1). The distance between two sampling sites was more than 1 km. The three replicated 1 l water samples were collected at each sampling site (Fig. 2). The eDNA was collected by 0.45 µm MCE membrane filtration (Pall Whatman, UK) from water samples and each membrane enriched with DNA was stored individually in a 2 ml centrifuge tube at -10℃ in the field. Meanwhile, 300 ml purified water was filtered to serve as a negative control in order to detect contamination.

### Step description

The eDNA was extracted using E.Z.N.A. Water DNA Kit and protocols of the kit were followed. Before DNA extraction, the experimental bench and equipment were regularly cleaned using 5% bleach and then 75% ethanol, in order to prevent cross-contamination. The extracted DNA samples were stored at -20°C for subsequent experiments. PCR amplification was performed using "MiFish-U" primer sets for multiple fish species detection (Forward: GTCGGTAAAACTCGTGCCAGC, Reverse: CATAGTGGGGTATCTAATCCCAGTTTG) (Miya et al. 2015). Both forward and reverse primers were tagged with oligonucleotide designed by the Shanghai Biozeron Biotechnology Co. Ltd, China and each sample had a specific tag (Table 1). A PCR system was total 25 µl volume, containing 4 µl of 5×FastPfu Buffer, 2 µl of 2.5 mM dNTPs, 0.8 µl Forward Primer (5 μM), 0.8 µl Reverse Primer(5 μM), 0.4 µl FastPfu Polymerase, 10 ng of Template DNA and 12 µl ddH^®^_2_O. The PCR reaction conditions were the following: pre-denaturation for 5 min at 95℃, followed by 32 cycles of denaturation (30 sec at 95℃), annealing (30 sec at 55℃) and elongation (45 sec at 72℃) and final elongation for 10 min at 72℃. And two negative controls (PCR blank) were set up in each PCR reaction, in order to monitor cross-contamination. The PCR products at each sampling site were verified at 2%. The libraries were built and then sequenced on an Illumina Novaseq platform (the Shanghai Biozeron Biotechnology Co. Ltd, China) using 150 bp paired-end sequencing.

The original sequences obtained from the Illumina Novaseq platform were initially processed using QIIME 2 software (Bolyen et al. 2019) and the end sequences demultiplexed, based on the tags and primers were removed from the end. The reads were then trimmed to 160-180 bp using CUTADAPT software (Martin 2011). Next, the data obtained by sequencing were spliced and filtered in order to obtain high-quality sequences, which were clustered into MOTUs using VSEARCH software with 97% similarity (Zhang et al. 2019). Finally, the sequences of MOTUs were aligned with NCBI-BLAST (https://www.ncbi.nlm.nih.gov/BLAST, version in March 2024) under the default parameter settings for taxonomic annotation (Djurhuus et al. 2017). We used the following criteria for taxonomic assignment: a) if the query sequence matched one locally occurring species in the NCBI database (https://www.ncbi.nlm.nih.gov) with ≥ 97% identity, the species was assigned, b) if the query sequence matched more than one locally occurring species in the NCBI with ≥ 97% identity, the lowest taxonomic level (i.e. genus or family) that contains all these species was assigned, c) if the query sequence matched a non-native species in the NCBI with ≥ 97% identity, meanwhile this non-native species belonged to the same genus as the known native species, this genus was assigned. The sequences assigned to “NA” or assigned to humans, birds, mammals or amphibians were removed. The geographical distribution of each species was verified by the Species Catalogue of China. Volume 2, Animals, Vertebrates. V (Zhang et al. 2020) and the FishBase database (http://www.fishbase.org/search.php).

## Geographic coverage

### Description

We surveyed six localities in the sea area surrounding WZZ (Fig. 1). The investigation involved nearly 249325.426 m² measured using ArcGIS 10.8 software.

### Coordinates

18.304 and 18.316 Latitude; 109.759 and 109.773 Longitude.

## Taxonomic coverage

### Description

In total, two classes, 17 orders, 63 families, 124 genera and 188 fish species (including nine undetermined species denoted by genus sp.), were detected using eDNA in the area surrounding WZZ.

### Taxa included

**Table:** 

Rank	Scientific Name	
class	Chondrichthyes	
class	Osteichthyes	
order	Anguilliformes	
order	Atheriniformes	
order	Aulopiformes	
order	Beloniformes	
order	Beryciformes	
order	Carcharhiniformes	
order	Clupeiformes	
order	Elopiformes	
order	Gadiformes	
order	Gasterosteiformes	
order	Mugiliformes	
order	Myliobatiformes	
order	Perciformes	
order	Pleuronectiformes	
order	Scorpaeniformes	
order	Siluriformes	
order	Tetraodontiformes	
family	Acanthuridae	
family	Acropomatidae	
family	Ambassidae	
family	Ammodytidae	
family	Apogonidae	
family	Atherinidae	
family	Balistidae	
family	Belonidae	
family	Blenniidae	
family	Bothidae	
family	Bregmacerotidae	
family	Caesionidae	
family	Callionymidae	
family	Carangidae	
family	Carcharhinidae	
family	Chaetodontidae	
family	Chirocentridae	
family	Cirrhitidae	
family	Clupeidae	
family	Cynoglossidae	
family	Elopidae	
family	Engraulidae	
family	Ephippidae	
family	Fistulariidae	
family	Gerreidae	
family	Gobiidae	
family	Haemulidae	
family	Hemiramphidae	
family	Holocentridae	
family	Kuhliidae	
family	Kyphosidae	
family	Labridae	
family	Latidae	
family	Leiognathidae	
family	Lethrinidae	
family	Lutjanidae	
family	Malacanthidae	
family	Monacanthidae	
family	Mugilidae	
family	Mullidae	
family	Muraenidae	
family	Myliobatidae	
family	Nemipteridae	
family	Ostraciidae	
family	Pempheridae	
family	Plesiopidae	
family	Plotosidae	
family	Pomacanthidae	
family	Pomacentridae	
family	Scaridae	
family	Scatophagidae	
family	Sciaenidae	
family	Scombridae	
family	Scorpaenidae	
family	Serranidae	
family	Siganidae	
family	Sillaginidae	
family	Sphyraenidae	
family	Synaphobranchidae	
family	Synodontidae	
family	Terapontidae	
family	Tetraodontidae	
family	Tripterygiidae	
genus	*Abudefduf*	
genus	*Acanthurus*	
genus	*Acentrogobius*	
genus	*Acropoma*	
genus	*Aetobatus*	
genus	*Alepes*	
genus	*Aluterus*	
genus	*Ambassis*	
genus	*Ammodytes*	
genus	*Anampses*	
genus	*Andamia*	
genus	*Arothron*	
genus	*Atherinomorus*	
genus	*Bathygobius*	
genus	*Blenniella*	
genus	*Branchiostegus*	
genus	*Bregmaceros*	
genus	*Caesio*	
genus	*Callionymus*	
genus	*Cantherhines*	
genus	*Caranx*	
genus	*Carcharhinus*	
genus	*Centropyge*	
genus	*Cephalopholis*	
genus	*Chaetodon*	
genus	*Cheilinus*	
genus	*Chelon*	
genus	*Chirocentrus*	
genus	*Chrysiptera*	
genus	*Cirrhitus*	
genus	*Cirripectes*	
genus	*Clupanodon*	
genus	*Collichthys*	
genus	*Coris*	
genus	*Cromileptes*	
genus	*Cynoglossus*	
genus	*Decapterus*	
genus	*Dendrophysa*	
genus	*Diagramma*	
genus	*Dysomma*	
genus	*Echidna*	
genus	*Ellochelon*	
genus	*Elops*	
genus	*Encrasicholina*	
genus	*Engraulis*	
genus	*Engyprosopon*	
genus	*Enneapterygius*	
genus	*Entomacrodus*	
genus	*Epinephelus*	
genus	*Exallias*	
genus	*Favonigobius*	
genus	*Fistularia*	
genus	*Gazza*	
genus	*Gerres*	
genus	*Glossogobius*	
genus	*Gymnomuraena*	
genus	*Gymnothorax*	
genus	*Halichoeres*	
genus	*Helcogramma*	
genus	*Hemigymnus*	
genus	*Herklotsichthys*	
genus	*Hypoatherina*	
genus	*Hyporhamphus*	
genus	*Istiblennius*	
genus	*Istigobius*	
genus	*Kuhlia*	
genus	*Kyphosus*	
genus	*Lagocephalus*	
genus	*Lates*	
genus	*Lethrinus*	
genus	*Lutjanus*	
genus	*Moolgarda*	
genus	*Mugil*	
genus	*Mugilogobius*	
genus	*Myripristis*	
genus	*Neopomacentrus*	
genus	*Nuchequula*	
genus	*Odontamblyopus*	
genus	*Odonus*	
genus	*Oedalechilus*	
genus	*Osteomugil*	
genus	*Ostorhinchus*	
genus	*Ostracion*	
genus	*Parablennius*	
genus	*Parascorpaena*	
genus	*Parupeneus*	
genus	*Pelates*	
genus	*Pempheris*	
genus	*Platax*	
genus	*Plectroglyphidodon*	
genus	*Plesiops*	
genus	*Plotosus*	
genus	*Pomacentrus*	
genus	*Pomadasys*	
genus	*Pseudobalistes*	
genus	*Pseudogobius*	
genus	*Pterocaesio*	
genus	*Rastrelliger*	
genus	*Rhinecanthus*	
genus	*Salarias*	
genus	*Sardinella*	
genus	*Sardinops*	
genus	*Saurida*	
genus	*Scarus*	
genus	*Scatophagus*	
genus	*Scolopsis*	
genus	*Scomber*	
genus	*Secutor*	
genus	*Selar*	
genus	*Siganus*	
genus	*Sillago*	
genus	*Sphyraena*	
genus	*Spratelloides*	
genus	*Stethojulis*	
genus	*Stolephorus*	
genus	*Terapon*	
genus	*Thalassoma*	
genus	*Thryssa*	
genus	*Thunnus*	
genus	*Trachinotus*	
genus	*Trachurus*	
genus	*Tylosurus*	
genus	*Upeneus*	
genus	*Zenarchopterus*	
species	*Abudefdufnotatus* (Day, 1870)	
species	*Abudefdufseptemfasciatus* (Cuvier, 1830)	
species	*Abudefdufsexfasciatus* (Lacépède, 1801)	
species	*Abudefdufsordidus* (Forsskål, 1775)	
species	*Abudefdufvaigiensis* (Quoy & Gaimard, 1825)	
species	*Acanthurustriostegus* (Linnaeus, 1758)	
species	*Acentrogobiusviganensis* (Steindachner, 1893)	
species	*Acropomajaponicum* Günther, 1859	
species	*Aetobatusnarinari* (Euphrasen, 1790)	
species	*Alepesdjedaba* (Forsskål, 1775)	
species	*Alepeskleinii* (Bloch, 1793)	
species	*Alepesvari* (Cuvier, 1833)	
species	*Aluterusscriptus* (Osbeck, 1765)	
species	*Ambassisurotaenia* Bleeker, 1852	
species	*Ammodytespersonatus* Girard, 1856	
species	*Anampsescaeruleopunctatus* Rüppell, 1829	
species	*Andamiatetradactylus* (Bleeker, 1858)	
species	*Arothronstellatus* (Bloch & Schneider, 1801)	
species	*Atherinomoruslacunosus* (Forster, 1801)	
species	*Atherinomorusregina* (Seale, 1910)	
species	*Bathygobiuscotticeps* (Steindachner, 1879)	
species	*Bathygobiushongkongensis* Lam, 1986	
species	*Blenniellabilitonensis* (Bleeker, 1858)	
species	*Branchiostegusargentatus* (Cuvier, 1830)	
species	*Bregmacerosmcclellandi* Thompson, 1840	
species	*Caesiocaerulaurea* Lacépède, 1801	
species	*Callionymusmeridionalis* Suwardji, 1965	
species	*Cantherhinespardalis* (Rüppell, 1837)	
species	*Caranxsexfasciatus* Quoy & Gaimard, 1825	
species	*Caranxtille* Cuvier, 1833	
species	*Carcharhinusmelanopterus* (Quoy & Gaimard, 1824)	
species	*Centropygevrolikii* (Bleeker, 1853)	
species	*Cephalopholisargus* Bloch & Schneider, 1801	
species	*Cephalopholisboenak* (Bloch, 1790)	
species	*Chaetodonauriga* Forsskål, 1775	
species	*Chaetodonplebeius* Cuvier, 1831	
species	*Chaetodonrafflesii* Anonymous [Bennett], 1830	
species	*Chelonaffinis* (Günther, 1861)	
species	*Chelonhaematocheilus* (Temminck & Schlegel, 1845)	
species	*Chelonmacrolepis* (Smith, 1846)	
species	*Chirocentrusdorab* (Forsskål, 1775)	
species	*Chrysipterabiocellata* (Quoy & Gaimard, 1825)	
species	*Chrysipterabrownriggii* (Bennett, 1828)	
species	*Chrysipteraglauca* (Cuvier, 1830)	
species	*Chrysipteraunimaculata* (Cuvier, 1830)	
species	*Cirrhituspinnulatus* (Forster, 1801)	
species	*Cirripectesimitator* Williams, 1985	
species	*Clupanodonthrissa* (Linnaeus, 1758)	
species	*Collichthyslucidus* (Richardson, 1844)	
species	*Corisgaimard* (Quoy & Gaimard, 1824)	
species	*Cromileptesaltivelis* (Valenciennes, 1828)	
species	*Cynoglossusrobustus* Günther, 1873	
species	*Decapterusmacrosoma* Bleeker, 1851	
species	*Decapterusmaruadsi* (Temminck & Schlegel, 1843)	
species	*Dendrophysarusselii* (Cuvier, 1829)	
species	*Diagrammamelanacrum* Johnson & Randall, 2001	
species	*Dysommaanguillare* Barnard, 1923	
species	*Echidnanebulosa* (Ahl, 1789)	
species	*Echidnapolyzona* (Richardson, 1845)	
species	*Ellochelonvaigiensis* (Quoy & Gaimard, 1825)	
species	*Elopsmachnata* (Forsskål, 1775)	
species	*Encrasicholinaheteroloba* (Rüppell, 1837)	
species	*Encrasicholinapunctifer* Fowler, 1938	
species	*Engyprosoponmultisquama* Amaoka, 1963	
species	*Enneapterygiusbahasa* Fricke, 1997	
species	*Enneapterygiusphilippinus* (Peters, 1868)	
species	*Entomacroduscaudofasciatus* (Regan, 1909)	
species	*Entomacrodusdecussatus* (Bleeker, 1858)	
species	*Entomacrodusstriatus* (Valenciennes, 1836)	
species	*Entomacrodusthalassinus* (Jordan & Seale, 1906)	
species	*Epinephelusfuscoguttatus* (Forsskål, 1775)	
species	*Epinephelusmultinotatus* (Peters, 1876)	
species	*Epinephelustrimaculatus* (Valenciennes, 1828)	
species	*Exalliasbrevis* (Kner, 1868)	
species	*Favonigobiusreichei* (Bleeker, 1854)	
species	*Fistulariacommersonii* Rüppell, 1838	
species	*Gazzaminuta* (Bloch, 1795)	
species	*Gerreserythrourus* (Bloch, 1791)	
species	*Gerresfilamentosus* Cuvier, 1829	
species	*Gerresoyena* (Forsskål, 1775)	
species	*Glossogobiuscelebius* (Valenciennes, 1837)	
species	*Gymnomuraenazebra* (Shaw, 1797)	
species	*Gymnothoraxchilospilus* Bleeker, 1864	
species	*Gymnothoraxfimbriatus* (Bennett, 1832)	
species	*Gymnothoraxflavimarginatus* (Rüppell, 1830)	
species	*Gymnothoraxkidako* (Temminck & Schlegel, 1846)	
species	*Gymnothoraxpictus* (Ahl, 1789)	
species	*Gymnothoraxundulatus* (Lacépède, 1803)	
species	*Halichoeresargus* (Bloch & Schneider, 1801)	
species	*Halichoeresmarginatus* Rüppell, 1835	
species	*Helcogrammafuscipectoris* (Fowler, 1946)	
species	*Hemigymnusmelapterus* (Bloch, 1791)	
species	*Herklotsichthysquadrimaculatus* (Rüppell, 1837)	
species	*Hypoatherinatemminckii* (Bleeker, 1854)	
species	*Hyporhamphusdussumieri* (Valenciennes, 1847)	
species	*Istiblenniusdussumieri* (Valenciennes, 1836)	
species	*Istiblenniusedentulus* (Forster & Schneider, 1801)	
species	*Istigobiusornatus* (Rüppell, 1830)	
species	*Kuhliamugil* (Forster, 1801)	
species	*Kyphosusbigibbus* Lacépède, 1801	
species	*Kyphosuscinerascens* (Forsskål, 1775)	
species	*Kyphosusvaigiensis* (Quoy & Gaimard, 1825)	
species	*Lagocephalusspadiceus* (Richardson, 1845)	
species	*Latescalcarifer* (Bloch, 1790)	
species	*Lethrinusatkinsoni* Seale, 1910	
species	*Lethrinusharak* (Forsskål, 1775)	
species	*Lethrinusnebulosus* (Forsskål, 1775)	
species	*Lethrinusornatus* Valenciennes, 1830	
species	*Lethrinusxanthochilus* Klunzinger, 1870	
species	*Lutjanusargentimaculatus* (Forsskål, 1775)	
species	*Lutjanusfulviflamma* (Forsskål, 1775)	
species	*Lutjanusmalabaricus* (Bloch & Schneider, 1801)	
species	*Lutjanusmonostigma* (Cuvier, 1828)	
species	*Lutjanusstellatus* Akazaki, 1983	
species	*Moolgardaseheli* (Forsskål, 1775)	
species	*Mugilcephalus* Linnaeus, 1758	
species	*Mugilogobiuschulae* (Smith, 1932)	
species	*Myripristiskuntee* Valenciennes, 1831	
species	*Neopomacentruscyanomos* (Bleeker, 1856)	
species	*Nuchequulanuchalis* (Temminck & Schlegel, 1845)	
species	*Odontamblyopuslacepedii* (Temminck & Schlegel, 1845)	
species	*Odonusniger* (Rüppell, 1836)	
species	*Oedalechiluslabiosus* (Valenciennes, 1836)	
species	*Osteomugilspeigleri* (Bleeker, 1858)	
species	*Ostorhinchuscookii* (Macleay, 1881)	
species	*Ostorhinchusfasciatus* (White, 1790)	
species	*Ostracioncubicus* Linnaeus, 1758	
species	*Parablenniusyatabei* (Jordan & Snyder, 1900)	
species	*Parascorpaenamossambica* (Peters, 1855)	
species	*Parupeneusciliatus* (Lacépède, 1802)	
species	*Pelatesquadrilineatus* (Bloch, 1790)	
species	*Pempherisadusta* Bleeker, 1877	
species	*Pempherisxanthoptera* Tominaga, 1963	
species	*Plataxteira* (Forsskål, 1775)	
species	*Plectroglyphidodondickii* (Liénard, 1839)	
species	*Plectroglyphidodonleucozonus* (Bleeker, 1859)	
species	*Plectroglyphidodonobreptus* (Whitley, 1948)	
species	*Plesiopscoeruleolineatus* Rüppell, 1835	
species	*Plotosuslineatus* (Thunberg, 1787)	
species	*Pomacentruschrysurus* Cuvier, 1830	
species	*Pomadasysmaculatus* (Bloch, 1793)	
species	*Pseudobalistesflavimarginatus* (Rüppell, 1829)	
species	*Pseudogobiusjavanicus* (Bleeker, 1856)	
species	*Pterocaesiodigramma* (Bleeker, 1864)	
species	*Rastrelligerkanagurta* (Cuvier, 1816)	
species	*Rhinecanthusaculeatus* (Linnaeus, 1758)	
species	*Salariasfasciatus* (Bloch, 1786)	
species	*Sardinellagibbosa* (Bleeker, 1849)	
species	*Sardinellalemuru* Bleeker, 1853	
species	*Sardinopssagax* (Jenyns, 1842)	
species	*Sauridaundosquamis* (Richardson, 1848)	
species	*Scaruspsittacus* Forsskål, 1775	
species	*Scarusrivulatus* Valenciennes, 1840	
species	*Scatophagusargus* (Linnaeus, 1766)	
species	*Scolopsisciliata* (Lacépède, 1802)	
species	*Scomberjaponicus* Houttuyn, 1782	
species	*Secutorruconius* (Hamilton, 1822)	
species	*Selarcrumenophthalmus* (Bloch, 1793)	
species	*Siganuscanaliculatus* (Park, 1797)	
species	*Siganusfuscescens* (Houttuyn, 1782)	
species	*Siganusguttatus* (Bloch, 1787)	
species	*Siganusspinus* (Linnaeus, 1758)	
species	*Sillagosihama* (Forsskål, 1775)	
species	*Sphyraenajello* Cuvier, 1829	
species	*Spratelloidesdelicatulus* (Bennett, 1832)	
species	*Spratelloidesgracilis* (Temminck & Schlegel, 1846)	
species	*Stethojulisbandanensis* (Bleeker, 1851)	
species	*Stethojulisterina* Jordan & Snyder, 1902	
species	*Stethojulistrilineata* (Bloch & Schneider, 1801)	
species	*Stolephoruswaitei* Jordan & Seale, 1926	
species	*Teraponjarbua* (Forsskål, 1775)	
species	*Thryssakammalensis* (Bleeker, 1849)	
species	*Thunnustonggol* (Bleeker, 1851)	
species	*Trachinotusbaillonii* (Lacépède, 1801)	
species	*Trachinotusovatus* (Linnaeus, 1758)	
species	*Trachurusjaponicus* (Temminck & Schlegel, 1844)	
species	*Tylosuruscrocodilus* (Péron & Lesueur, 1821)	
species	*Upeneusjaponicus* (Houttuyn, 1782)	
species	*Zenarchopterusdunckeri* Mohr, 1926	
species	*Cheilinus* sp.	
species	*Engraulis* sp.	
species	*Enneapterygius* sp.	
species	*Gymnomuraena* sp.	
species	*Ostorhinchus* sp.	
species	*Platax* sp.	
species	*Sardinella* sp.	
species	*Siganus* sp.	
species	*Thalassoma* sp.	

## Temporal coverage

**Data range:** 2022-6-26 – 2022-6-27; 2023-1-15 – 2023-1-17; 2023-9-06 – 2023-9-08.

## Usage licence

### Usage licence

Creative Commons Public Domain Waiver (CC-Zero)

## Data resources

### Data package title

Fish taxon-occurrences surrounding WZZ, Hainan Province, China based on eDNA

### Number of data sets

2

### Data set 1.

#### Data set name

Fish taxon-occurrences surrounding WZZ detected by eDNA

#### Data format

Darwin Core

#### Description

The dataset presents the results of 188 fish species detected by eDNA at six sampling localities surrounding WZZ and includes the latitude and longitude information of the sampling sites. Important information including the taxonomic, geographic location of the occurrence and habitat condition was provided (Suppl. material 1).

**Data set 1.** 

Column label	Column description
occurrenceID	Unique occurrence identifier.
scientificName	The full scientific name.
kingdom	The full scientific name of the kingdom in which the taxon is classified.
Phylum	The full scientific name of the phylum or division in which the taxon is classified.
Class	The full scientific name of the class in which the taxon is classified.
Order	The full scientific name of the order in which the taxon is classified.
Family	The full scientific name of the family in which the taxon is classified.
Genus	The full scientific name of the genus in which the taxon is classified.
taxonRank	The taxonomic rank of the most specific name in the scientificName as it appears in the original record.
locality	The specific description of the county from where specimens are collected.
county	The full, unabbreviated name of the next smaller administrative region than stateProvince (county, shire, department, etc.) in which the Location occurs.
stateProvince	The name of the next smallest administrative region than country (state, province, canton, department, region etc.) in which the Location occurs.
Country	The full, unabbreviated name of the country where the organism was collected.
waterBody	The name of the water body in which the Location occurs.
habitat	A category or description of the habitat in which the Event occurred.
locationID	A spatial region or named place. The locationID refers to serial number of each sampling site in this study.
decimalLatitude	The geographic latitude (in decimal degrees, using the spatial reference system given in geodeticDatum) of the geographic centre of a Location.
decimalLongitude	The geographic longitude (in decimal degrees, using the spatial reference system given in geodeticDatum) of the geographic centre of a Location.
geodeticDatum	The geographic information system (GIS) upon which the geographic coordinates given in decimalLatitude, decimalLongitude and meterElevation are based.
basisOfRecord	The specific nature of the data record.
eventDate	The date-time or interval during which a dwc:Event occurred. For occurrences, this is the date-time when the dwc:Event was recorded. Not suitable for a time in a geological context.
samplingProtocol	The names of, references to, or descriptions of the methods or protocols used during a dwc:Event.

### Data set 2.

#### Data set name

MOTUs information of fish species detected by eDNA

#### Data format

Darwin Core

#### Description

The dataset presents the nucleotides sequence, sequences source and abundance of sequences of each MOTU, as well as its matched species in NCBI (https://www.ncbi.nlm.nih.gov/BLAST) and the best NCBI BLAST sequence similarity, obtained through high-throughput sequencing, based eDNA samples collected from the sea area surrounding WZZ (Suppl. material 2).

**Data set 2.** 

Column label	Column description
scientificName	The full scientific name.
associatedSequences	A list (concatenated and separated) of identifiers (publication, global unique identifier, URI) of genetic sequence information associated with the Occurrence. The associatedSequences refers to MOTUs sequences of each scientificName.
organismQuantity	A number or enumeration value for the quantity of organisms.
organismQuantityType	The type of quantification system used for the quantity of organisms.
dateIdentified	The date on which the subject was determined as representing the Taxon.
identificationReferences	A list (concatenated and separated) of references (publication, global unique identifier, URI) used in the Identification.
identificationRemarks	Comments or notes about the Identification.

## Additional information

**Figure 3.**
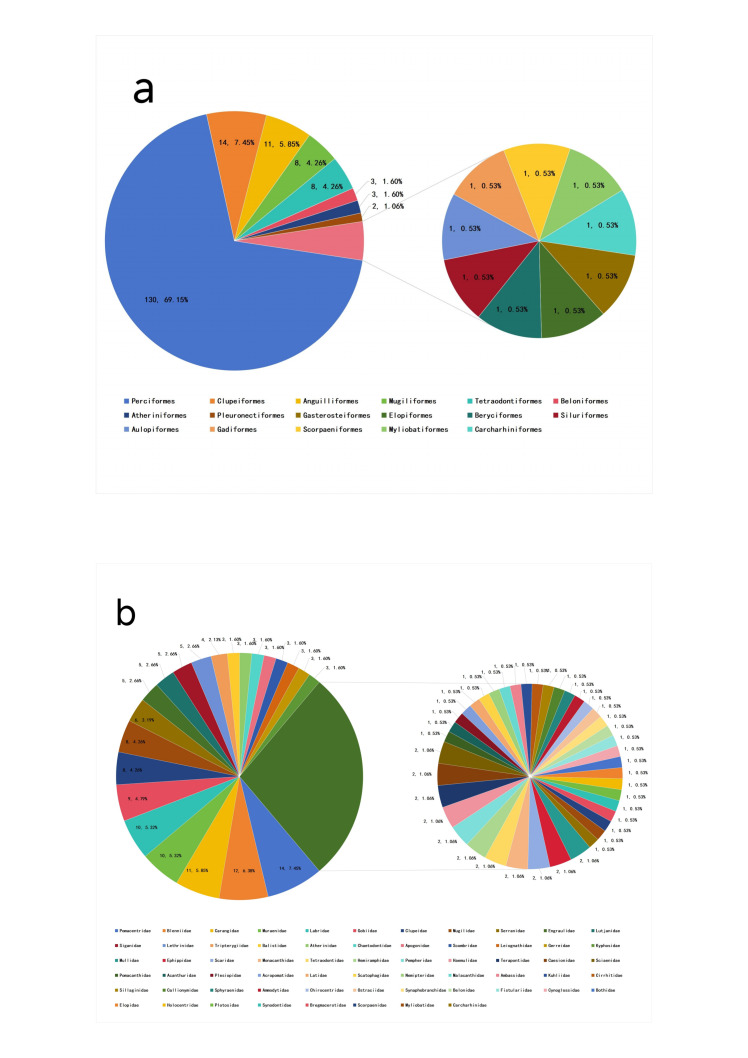
The composition of fish fauna surrounding WZZ at the order level (a) and family level (b).

A total of 188 fish species (including nine undetermined species denoted by genus sp.) were detected using eDNA surrounding WZZ and fish fauna was analysed (Fig. 3). At the order level, Perciformes included the most species (n = 130), accounting for 69.15% of the total number of fish species detected and occupied absolute advantage compared to other orders. The Clupeiformes (n = 14) was the second largest order, accounting for 7.45%, followed by Anguilliformes (n = 11) accounting for 5.85%. Additionally, the Mugiliformes and Tetraodontiformes both had eight species, accounting for 4.26%, respectively. The Atheriniformes and Beloniformes both included three species, separately accounting for 1.60%, as well as the Pleuronectiformes containing two species and accounting for 1.06%. The rest of the orders, Aulopiformes, Beryciformes, Carcharhiniformes, Elopiformes, Gadiformes, Gasterosteiformes, Myliobatiformes, Scorpaeniformes and Siluriformes, all harboured only one species and 0.53% of the total number (Fig. 3). At the family level, the most dominant was Pomacentridae and it included 14 species, accounting for 7.45% of the total number. The second family is Blenniidae (n = 12, 6.38%), followed by Carangidae (n = 11, 5.85%). Most of the families had only one species (Fig. 3).

We compared the fish species richness detected by eDNA to that by fishing nets, in order to assess the effects of eDNA on species identification. A total of 115 fish species belonging to 11 orders and 49 families, as well as a total of 174 fish species belonging to two classes, 12 orders, 65 families and 123 genera were investigated at WZZ through trawl nets in 2019 and 2020-2021, respectively (Wang et al. 2021, Luo et al. 2023). According to our results, the eDNA could supply more information of fish species, compared to traditional fishing nets. Meanwhile, the differences between the two monitoring methods in the number of family and order may be related to seasonal changes of the marine fish community. Although some species cannot be annotated using the NCBI database or only be identified at the genus level, based on MOTUs sequences, the eDNA datasets established in this study can help us to solve this problem to a certain extent.

## Supplementary Material

BFB8264B-593D-509C-937C-CAE83427F8EB 10.3897/BDJ.12.e127120.suppl1 Supplementary material 1 Fish taxon-occurrences surrounding WZZ, Hainan Province, China based on eDNA Data type dataset Brief description The dataset presents the results of 188 fish species detected by eDNA at six sampling localities surrounding WZZ, also above the latitude and longitude information of the sampling sites. The important information including taxonomic, geographic location of the occurrence and habitat condition was provided. File: oo_1052555.csv https://binary.pensoft.net/file/1052555 Chongzhao Wang, Zhenhua Ma, Kun Cao, Ting Jiang, Rui Yang, Rui Xi, Xin Wang, Yingchun Xing

34E390CB-1098-5B7D-902B-E87628DF5A38 10.3897/BDJ.12.e127120.suppl2 Supplementary material 2 MOTUs information of marine fish detected by eDNA Data type dataset Brief description The dataset presents the nucleotides sequence, source of sequences and abundance of sequences of each MOTU, as well as its matched species in NCBI (https://www.ncbi.nlm.nih.gov/BLAST) and the best NCBI BLAST sequence similarity, obtained through high-throughput sequencing based eDNA samples collected from the area surrounding WZZ. File: oo_1043351.csv https://binary.pensoft.net/file/1043351 Chongzhao Wang, Zhenhua Ma, Kun Cao, Ting Jiang, Rui Yang, Rui Xi, Xin Wang, Yingchun Xing

## Figures and Tables

**Table 1.** The oligo tag sequences corresponding to the samples.

Sample ID	Forward Oligo Tags	Reverse Oligo Tags
2023-WZZD-1	TAACGA	CGCTT
2023-WZZD-2	TAACGA	GCCAGT
2023-WZZD-3	TAACGA	TCTCAGTC
2023-WZZD-4	TAACGA	CGCTGAT
2023-WZZD-5	AACCGAGA	TCACC
2023-WZZD-6	AACCGAGA	ATGCCT
2023-WZZD-NTC	AACCGAGA	CGCTT
